# The J‐ and G/F‐domains of the major *Synechocystis* DnaJ protein Sll0897 are sufficient for cell viability but not for heat resistance

**DOI:** 10.1002/2211-5463.12980

**Published:** 2020-09-27

**Authors:** Eva Düppre, Dirk Schneider

**Affiliations:** ^1^ Department of Chemistry, Biochemistry Johannes Gutenberg University Mainz Germany

**Keywords:** chaperone, cyanobacteria, DnaJ, Hsp40, stress response, *Synechocystis*

## Abstract

Hsp70 proteins and their Hsp40 co‐chaperones are essential components of cellular chaperone networks in both prokaryotes and eukaryotes. Here, we performed a genetic analysis to define the protein domains required for the key functions of the major Hsp40/DnaJ protein Sll0897 of the cyanobacterium *Synechocystis* sp. PCC6803. The expression of the N‐terminally located J‐ and G/F‐domains is essential and sufficient for the proteins’ fundamental *in vivo* functions, whereas the presence of the full‐length protein, containing the C‐terminal substrate‐binding domains, is crucial under stress conditions.

## Graphical abstract

Here, we show that the expression of the main DnaJ protein encoded in the cyanobacterium *Synechocystis* sp. PCC6803 is essential for cell survival. While the N‐terminally located J‐ and G/F‐domains are sufficient for the protein to fulfill its fundamental *in vivo* functions, the presence of the remaining domains is vital under stress conditions.

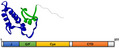

**Abbreviations**
CTD: C‐terminal domain
G/F region: glycine‐ and phenylalanine‐rich region
HSP: heat shock protein
wt: wild‐type

Hsp70 proteins and their Hsp40 co‐chaperones are essential components of cellular chaperone networks in both prokaryotes and eukaryotes [1, 2]. Hsp70s, which are named DnaK in bacteria, are involved in the folding of newly synthesized proteins and in assembly and reorganization of protein complexes, and assist in protein targeting within cells and protein translocation across cellular membranes [2, 3]. The interaction of Hsp70s with protein clients is controlled by ATP‐binding and hydrolysis, and two co‐chaperones, an ATP exchange factor (named GrpE in bacteria) and Hsp40 proteins containing a J‐domain, typically control the chaperone activity of Hsp70s. The J‐domain containing Hsp40 co‐chaperone is also known as J‐protein or DnaJ. Hsp40s couple substrate‐binding to Hsp70 with ATP hydrolysis, thereby assisting in efficiently trapping Hsp70 substrates. Hsp40s can bind protein substrates independently, deliver substrates to Hsp70s, and may control how substrates are being recognized and how they initially interact with Hsp70s [4].

Structurally, all members of the Hsp40 family share a 60‐ to 70‐amino‐acid‐long J‐domain, which binds to the nucleotide‐binding domain and to the substrate‐binding domain of Hsp70s [5]. Besides the conserved J‐domain, J‐domain proteins can contain additional protein domains (compare Fig. 1A). Based on a historical classification, Hsp40s proteins are grouped into three classes depending on the number of additional domains the individual proteins share with the prototypical J‐domain protein, the *E. coli* DnaJ. In class A proteins, a region rich in glycine and phenylalanine (G/F‐region) flanks the N‐terminal J‐domain and links it to a cysteine‐rich, zinc‐binding region and a conserved, large C‐terminal domain (CTD) that contains two beta domains followed by a short dimerization domain [6]. Class A DnaJ proteins can act as Hsp70 co‐chaperones, yet they also bind substrate proteins independently and prevent protein aggregation [7]. Noteworthy, bacterial class A proteins are highly conserved and all have a size comparable to the canonical *E. coli* DnaJ [4]. Class B proteins contain the J‐domain, the G/F‐region and the CTD, but always lack the cysteine‐rich, zinc‐binding region. Class C proteins solely contain the J‐domain, but can contain various additional domains involved in protein interaction and/or localization [8]. The CTD appears to be the major substrate‐binding region of J‐domain proteins. Consequently, while interaction with Hsp70s and stimulation of the Hsp70’s ATPase activity are conserved features of all proteins containing a J‐domain, most likely only class A‐ and class B DnaJ proteins are involved in substrate delivery. Class C proteins potentially assist in specifically localizing Hsp70s within a defined cellular compartment [4].

**Fig. 1:**
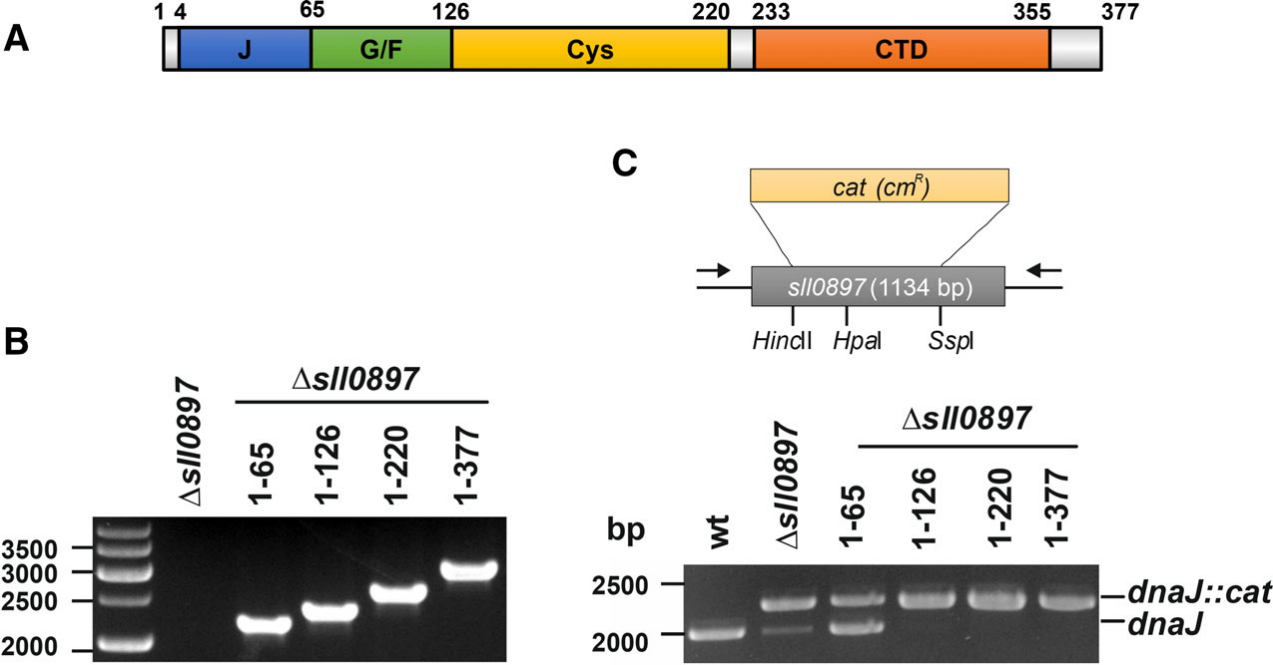
Expression of truncated Sll0897 in *Synechocystis* sp. PCC 6803 cells. (A) Domain structure of the Sll0897 protein containing the J‐, the glycine‐ and phenylalanine‐rich (G/F), the cysteine‐rich (Cys), and the C‐terminal domain (CTD). The amino acid positions, at which the respective domains start and end, are indicated. (B) PCR analysis of the full‐length and truncated *sll0897* genes integrated into the genome of a *Synechocystis* Δ*sll0897* strain at a neutral site. The neutral site (*slr0168*) encodes a hypothetical protein that is deleted when a foreign gene is integrated [21]. The integrated regions were amplified via PCR using DNA of the respective mutant strains as a template. All genes are part of the *Synechocystis* genome. (C) Deletion of the *Synechocystis sll0897* gene. Upper part: A major part of *sll0897* was deleted via integration of a chloramphenicol resistance cassette (*cat*) in the *Hinc*II and *Ssp*I restriction‐digested *sll0897* gene. Arrows indicate the binding sites of the primers used for the PCR analysis. Lower part: PCR analysis of the generated *Synechocystis* Δ*sll0897* strains. The *sll0897* region was amplified from the respective mutant strain. As a control, the wt *sll0897* gene was amplified from genomic wt DNA. The interrupted *sll0897* (sll0897*::cat*) was approximately 280 bp larger than the wt fragments. As a control, wt DNA was used for PCR analysis

The currently best characterized Hsp70 and Hsp40 proteins are still the prototypical DnaK and DnaJ proteins of the bacterium *E. coli* [1, 9]. Yet, in *E. coli* two additional Hsp70s are functionally expressed (HscA and HscC) besides the canonical DnaK protein, as well as one class B (CbpA) and four class C (HscB, DjlA, DjlB, and DjlC) J‐domain proteins additional to the sole class A DnaJ [1, 4, 9]. Defined interactions and cellular functions of specific DnaK/DnaJ interactions have been studied to a great extent in the *E. coli* system in recent decades [4]. Yet, while most organisms, from bacteria to men, contain multiple DnaK/Hsp70 and DnaJ/Hsp40 proteins, the interaction of defined partners and the physiological relevance of a Hsp70/Hsp40 chaperone network, plus the physiological significance of individual (co‐)chaperone proteins, are mostly only sparsely understood.

Cyanobacteria typically encode a single GrpE nucleotide exchange factor [10], but multiple DnaK and DnaJ proteins. The resulting chaperone network has been analyzed to some extent in the cyanobacteria *Synechococcus* sp. PCC 7942 [11, 12, 13, 14] and *Synechocystis* sp. PCC 6803 [15, 16, 17, 18]. Out of the three *Synechocystis* DnaK proteins, only DnaK1 (Sll0058) appears to be dispensable and the encoding gene can be deleted, whereas the two proteins DnaK2 (Sll0179) and DnaK3 (Sll1932) are essential [18]. While in *Synechocystis* at least seven DnaJ co‐chaperones are encoded, only the DnaJ protein encoded by the *orf sll0897* represents a true class A DnaJ protein and has been classified as the major DnaJ protein involved in stress response in *Synechocystis* [15]. Furthermore, two class B (Slr0093 and Sll1933) and four class C DnaJ proteins (Sll0909, Sll1011, Sll1384, and Sll1666) are functionally expressed in *Synechocystis* [15]. However, the observation that solely the class B DnaJ Sll1933 appears to be absolutely essential, whereas the deletion of the gene encoding the only class A protein Sll0897 was possible, was unexpected, as, for example, the expression of the canonical *Synechocystis* DnaK2 protein is essential [18].

Here, we show that the expression of a Sll0897 protein containing at least the J‐domain and the G/F‐domain is required and sufficient for the survival of *Synechocystis* cells at normal growth conditions. At elevated temperatures, the truncated protein cannot complement the growth defect, and thus, under stress conditions, the full‐length Sll0897 protein is required for cell growth. Hence, besides the before already identified class B DnaJ protein Sll1933, the class A protein Sll0897 is also essential in the cyanobacterium *Synechocystis* sp. PCC 6803.

## Methods

### Cell growth

*Synechocystis* sp. PCC 6803 wild‐type (wt) was cultivated photoautotrophically in liquid BG11 medium [19] at 34 °C under 33 μE m^−2^ s^−1^ of fluorescent cold white light. For monitoring the growth of the mutant strains, cells were diluted to an OD_750_ of 0.05 in BG11 medium. Photoautotrophic growth of the cultures at normal (34 °C) or under heat‐stress conditions (42 °C) was monitored via following the OD_750_ until the stationary phase was reached.

### Deletion of sll0897 and construction of *Synechocystis* strains expressing truncated Sll0897 proteins

For deletion, the *dnaJ* gene *sll0897* was amplified by PCR using the primers 5’*sll0897* and 3’*sll0897* (Table 1) and genomic *Synechocystis* wt DNA as a template. The PCR fragment was ligated into the plasmid pGEM^®^‐T Easy (Promega). A significant section of the *sll0897* gene was cut out of the plasmid by restriction digestion using *Hinc*II and *Spe*I, and a chloramphenicol resistance cassette (*cat*), derived from the plasmid pACYC184 (New England Biolabs, Frankfurt, Germany), was inserted into the gene. The resulting plasmid was transformed into *Synechocystis* wt cells [20], and positive clones, which have replaced the wt *sll0897* gene by the *cat* cassette, were selected on BG11 agar plates containing 10 μg mL^−1^ chloramphenicol. Individual colonies were continuously transferred onto selective agar plates containing increasing concentrations of chloramphenicol (up to 150 μg mL^‐1^) for more than one year. Complete segregation was tested via PCR using the primers 5’*sll0897* + and 3’*sll0897*+ (Table 1), which resulted in amplification of the *sll0897* genomic region including up‐ and downstream sequences.

**Table 1:** Primers used to amplify the full‐length and truncated *dnaJ sll0897* genes. Sequences are given in 5´→ 3´direction

Gene/genomic region	5’‐primer	3’‐primer
*sll0897*	ataggccatatgcctggggattattaccaaac	atacgaagctttatttatggaataatccccctaaaaatc
*sll0897*+	aaaatctcagcaccccagaaa	aaaagtggggcaaaaggtcat
pILAseqcheck	ggaagggggaattgtaacagc	gtcaaaggcaatctgttggg
1–65	ataggccatatgcctggggattattaccaaac	tagactagtttaggcaaaatcgcccatattgcc
1–126	ataggccatatgcctggggattattaccaaac	tagactagtttactcacagacctgacaggattc
1–220	ataggccatatgcctggggattattaccaaac	tagactagtttaccgtaaaccagcatctccttc
1–377	ataggccatatgcctggggattattaccaaac	tagactagtgctcatttatggaataatcccc

Fragments of the *sll0897* gene, coding for the individual DnaJ protein domains (compare Fig. 1), were amplified by PCR using the primers listed in Table 1, and the PCR products were ligated into the *Pst*I and *Xba*I restriction‐digested pILA plasmid [21]. The resulting constructs were transformed into *Synechocystis* cells, and positive clones were selected on BG11 agar plates containing 10 µg/mL kanamycin. After a continuous selection of increasing kanamycin concentrations, complete segregation was confirmed via PCR using the 5’pILAseqcheck and 3’pILAseqcheck primers (Table 1).

## Results and Discussion

### *s* *ll0897* is an essential dnaJ gene in *Synechocystis*

The previous conclusion that the *sll0897* gene is not essential in *Synechocystis* sp. PCC 6803 was unexpected as the encoded DnaJ protein Sll0897 is the only canonical class A DnaJ protein in *Synechocystis* and the canonical DnaK2 chaperone is essential in *Synechocystis*. While at least six other DnaJ proteins are expressed in *Synechocystis,* Sll0897 appears to be the major DnaJ representative in *Synechocystis* sp. PCC 6803 [15]. Interruption of *sll0897* resulted in impaired cell growth already under normal growth conditions and in a significantly reduced growth under heat‐stress conditions [15]. However, as the *sll0897* gene was interrupted by inserting a chloramphenicol resistance cassette into the single *Hpa*I site within the gene (compare Fig. 1C), it was still possible that an N‐terminal fragment, including the J‐ and the G/F‐domains, was produced in the mutant strain. In fact, the N‐terminal 108‐amino acids of the canonical *E. coli* DnaJ protein, containing the J‐domain and the G/F‐rich region, are sufficient to partially support the DnaK *in vivo* function [22].

Thus, to test whether the deletion of the almost complete *sll0897* gene is possible in *Synechocystis*, we deleted a 1184‐bp region of the *sll0897* gene and inserted a chloramphenicol resistance cassette (*cat*, Fig. 1C), ensuring that the expression of even the complete N‐terminal J‐domain was abolished. After transformation, the resistance cassette was integrated into the *Synechocystis* genome via homologous recombination, resulting in the deletion of the almost complete *sll0897* gene. However, as *Synechocystis* contains multiple identical genome copies [23], a chloramphenicol‐resistant *Synechocystis* strain does not necessarily have to have all genomic copies replaced by the deleted gene region. Therefore, transformed *Synechocystis* cells were cultivated on selective medium in the presence of increasing chloramphenicol concentrations, and the complete segregation of the strain was tested via PCR. Even after growing the cells for a prolonged time on selective medium, the complete deletion of the *sll0897* gene was not achieved. As can be seen in Fig. 1C, while a PCR fragment, corresponding in size to the *sll0897* genomic region with part of the original sequence replaced by the *cat* cassette, was observed in the deletion strain, a fragment corresponding in size to the wt gene was also still amplified. Thus, while the *sll0897* gene was deleted in some genomic copies, the complete deletion of the gene was not possible, indicating an essential physiological function of the encoded DnaJ protein Sll0897. Yet, to exclude potential polar effects caused by introducing the *cat* cassette, we subsequently inserted the *sll0897* wt gene for expression together with its natural promoter into a neutral site within the *Synechocystis* genome. Thereafter, the complete deletion of the endogenous *sll0897* was tested.

As can be seen in Fig. 1B, the full‐length *sll0897* wt gene (construct '1‐377') was successfully integrated into the *Synechocystis* genome, thereby allowing the expression of the Sll0897 full‐length protein. The full‐length protein encoded at the neutral site of the chromosome can replace the function of the endogenous (original) wt Sll0897 protein. Consequently, the complete deletion of the original *sll0897* gene was observed in this strain, excluding polar effects (Fig. 1C). Thus, the *sll0897* gene clearly is essential and cannot be completely deleted in *Synechocystis*.

### The Sll0897 J‐ and G/F‐domains are essential in *Synechocystis*

**Fig. 2:**
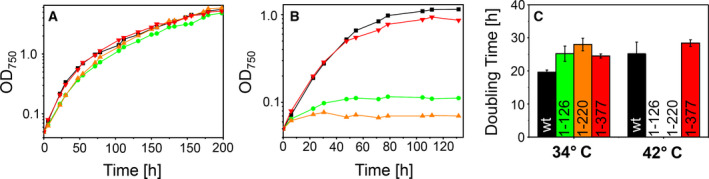
The DnaJ1 CTD is essential for heat sensitivity. The growth of the *Synechocystis* strains, expressing the wt or truncated Sll0897 proteins, at normal (34 °C, A) or elevated (42 °C, B) temperatures. Black: WT, green: Δsll0897 + 1‐126, orange: Δsll0897 + 1‐220, and red: Δsll0897 + 1‐377. The strains expressing only the 1‐126 and 1‐220 fragments stopped growing approximately 20 h after shifting the cells from 34 °C to 42 °C. (C) Doubling times of the individual strains calculated from curves shown in (A) and (B). Growth curves as shown in (A, B) were determined at least three times, and error bars (SD) are shown

The results presented above indicate that the previously reported interruption of *sll0897* might not have resulted in completely abolished expression of part of the *sll0897* gene, but rather potentially allowed cellular production of the N‐terminal Sll0897 domains, which was sufficient for cell survival. Thus, we next analyzed which Sll0897 domains are minimally required for survival of *Synechocystis* cells. Therefore, truncated genes coding for truncated Sll0897 proteins were inserted into the neutral site within the *Synechocystis* genome, and gene expression was controlled by the natural *sll0897* promoter. Thereafter, the complete deletion of the endogenous *sll0897* was tested. As can be seen in Fig. 1B, all truncated *sll0897* genes were successfully integrated into the *Synechocystis* genome, allowing cellular production of the encoded truncated Sll0897 proteins. Importantly, only if a protein or protein fragment expressed from the neutral site can replace the function of the wt Sll0897 protein, the complete deletion of the original *sll0897* gene is possible. Solely in the strain, in which a Sll0897 fragment containing exclusively the J‐domain is encoded (Sll0897_1–65_), two PCR products were detected, which correspond in size to the wt *sll0897* gene and to the deleted genomic region (Fig. 2C). Thus, encoding exclusively the DnaJ J‐domain at a different genomic locus cannot compensate for the deletion of the wt *sll0897* gene. Yet, the complete deletion of the wt *sll0897* gene was observed in strains where truncated Sll0897 versions are encoded, which contain at least the J‐ and the G/F‐domains (Fig. 1C). PCR analyses of these strains clearly showed only a single PCR product with a size of about 2275 bp, demonstrating the complete deletion of *sll0897*. No PCR fragment, corresponding in size to the wt *sll0897* gene (2006 bp), was amplified when genomic DNA isolated from these strains was used as a template.

Taken together, Sll0897 DnaJ proteins containing at least the J‐ and G/F‐domains need to be encoded for survival of *Synechocystis* cells.

### The C‐terminal domain of Sll0897 is crucial for heat sensitivity

While the above presented results show that Sll0897 fragments need to contain at least the J‐ and G/F‐domains for viability of *Synechocystis* cells, we next analyzed whether the expression of truncated DnaJ proteins still results in heat sensitivity.

At normal growth temperatures (34 °C), we observed growths of all generated strains, nearly identical to the growth of the wt strain (Fig. 2A). However, under heat‐stress conditions (42 °C), the strains encoding exclusively the Sll0897 J‐ and G/F‐domains (Sll0897_1–126_), as well as the strain in which the Sll0897 cysteine‐rich region is encoded in addition (Sll0897_1–220_), stopped growing approximately 20 hours after increasing the temperature (Fig. 2B). Noteworthy, when the full‐length *sll0897* gene was expressed from a different locus (Sll0897_1–377_), the generated deletion strain grew as well as the wt strain. The doubling times calculated from the growth curves shown in (A) and (B) are summarized in Fig. 2C. The wt and the Sll0897_1–377_ showed only slightly increased doubling times at 34 °C and at 42 °C. In contrast, while the Sll0897_1–126_ and Sll0897_1–220_ strains had doubling times comparable to the wt strain at 34 °C, at 42 °C the initial doubling times were significantly increased to more than 60 h before the cultures stopped growing completely (Fig. 2B). Therefore, we refrained from presenting doubling times. Thus, the cellular production of a Sll0897 protein containing solely the J‐ and G/F‐domains is crucial but not sufficient for viability of *Synechocystis* cells at elevated temperatures.

### The DnaJ J‐ and G/F‐domains are sufficient for cell viability but not for heat resistance

In contrast to *E. coli* or *Saccharomyces cerevisiae,* where Δ*dnaJ* cells are viable under non‐stress conditions [24, 25, 26, 27, 28], the here presented results show that the *sll0897* gene is essential in *Synechocystis*. The expression of at least the J‐ and G/F‐domains is crucial for viability and cell survival, albeit the cells were no longer heat‐resistant and showed a strong growth defect at elevated temperatures. Thus, the J‐ and G/F‐domains are sufficient for cell survival at normal growth temperatures but not under stress conditions.

While the J‐domain is responsible for stimulating the ATPase activity of DnaK proteins, both, the J‐ and G/F‐domains, interact with DnaK [29], and, in fact, in *E. coli* and in *Saccharomyces cerevisiae* the expression of a DnaJ fragment containing the J‐domain and the G/F‐rich region is minimally required to at least partially support the DnaK chaperone activity [22, 30, 31]. Thus, under normal growth conditions, the interaction of the Sll0897 J‐ and G/F‐domains with *Synechocystis* DnaK proteins appears to be crucial but sufficient for cell survival, as the expression of the truncated Sll0897_1–126_ protein resulted in normal growth. However, while several DnaJ proteins are functionally expressed in *Synechocystis* besides Sll0897 [15], the remaining DnaJs appear to neither be able to rescue the deleterious defects associated with a complete *sll0897* deletion, nor be able to complement the growth defect observed at elevated temperatures when only a truncated Sll0897 protein is encoded. As the two other class B DnaJ proteins in *Synechocystis* also contain a G/F‐region next to their J‐domain, our results indicate a unique function of the specific Sll0897 J‐ and G/F‐domains, which cannot be (fully) accomplished by any other DnaJ protein in *Synechocystis*. Only the expression of a Sll0897 protein, carrying the cysteine‐rich and the C‐terminal domain in addition to the J‐ and G/F‐domains, permitted cell growth at elevated temperatures (Fig. 2B). Most likely, binding of defined substrates to Sll0897 is vital at elevated temperatures, as the cysteine‐rich region and the CTD are involved in substrate binding by DnaJs [32]. However, we can also not exclude that truncation has destabilized the Sll0897 protein, limiting proper interaction with DnaK at elevated temperatures.

Together, in the present study we observed that the J‐ and G/F‐domains of cyanobacterial class A DnaJ proteins are absolutely required but sufficient for the fundamental DnaJ functions, whereas the C‐terminally located substrate‐binding domains are vital only under stress conditions, such as elevated temperatures.

## Author contributions

ED and DS conceptualized the data, visualized the data, and validated the data. ED was involved in methodology and formal analysis and investigated the data; and DS provided resources, wrote the manuscript, supervised the data, administrated the project, and acquired funding. All authors have read and agreed to the published version of the manuscript.

## Conflicts of interest

The authors declare no conflict of interest.

## Data Availability

The data are available from the corresponding author upon reasonable request.
